# Treatment of Hemorrhoids

**Published:** 1892-06

**Authors:** 


TREATMENT OF HEMORRHOIDS.—Dr. J. Brindley James writes to the *British Medical Journal* of February, 20, 1892, that he has for some years been in the habit of treating hemorrhoids by the simple process of applying calomel to them with the finger alone, and without a single exception he has done so with marked success, especially when inflammatory action was obvious in the hemorrhoidal mass, characterized by mucous discharge and hemorrhage, accompanied by most painful sensation of weight in the rectal region. All these symptoms under this simple influence were speedily relieved, with the still more important subsequent advantage of the patient's restoration to ease. "Only a few days ago," he writes, "a patient came to me suffering so acutely that he could neither sit nor walk freely, each movement of the body entailing exquisite pain. I have now seen him thoroughly enabled to pursue his usual occupation in happy immunity from these distressing symptoms."—*Medical Record.*

